# Effect of MELD-Na score on overall survival of periampullary cancer

**DOI:** 10.1007/s13304-024-01856-w

**Published:** 2024-05-07

**Authors:** Serkan Yılmaz, Mesut Yur

**Affiliations:** 1 Department of Surgical Oncology, Fethi Sekin State Hospital, Elazığ, Turkey; 2 Department of Surgical Oncology, Fırat University School of Medicine, 23280 Elazığ, Turkey

**Keywords:** MELD-Na score, Pancreatic adenocarcinoma, Pancreaticoduodenectomy

## Abstract

Periampullary cancers have a short overall survival (OS), and many prognostic factors have been studied for this purpose. They usually present with biliary obstruction, which negatively affects the liver, kidney, immune system, and cardiovascular system. This study aimed to investigate the effect of MELD-Na scores on OS in patients undergoing pancreaticoduodenectomy due to periampullary cancer. Patients who underwent pancreaticoduodenectomy due to periampullary cancer between January 2010 and January 2021 were included in the study. After applying the exclusion criteria, 80 of the 124 patients were included in the study. The demographic, laboratory, and pathologic data of the patients were analyzed retrospectively. Univariate analysis showed that MELD-Na score at admission, age-adjusted Charlson Comorbidity Index, adjuvant treatment, portal vein resection, lymphovascular invasion (LVI), T-stage, and tumor location were significantly associated with OS (*p* < 0.1). In multivariate analysis, MELD-Na score at admission (HR: 1.051, 95% CI [1.004–1.101]; *p* = 0.033), adjuvant treatment (HR: 4.717, 95% CI [2.371–9.383]; *p* < 0.001), LVI (HR: 2.473, 95% CI [1.355–4.515]; *p* = 0.003), and tumor location (HR: 2.380, 95% CI [1.274–4.445]; *p* = 0.007) were independent risk factors for OS. MELD-Na score, adjuvant treatment, LVI, and tumor location were independent risk factors for the OS of periampullary cancer. The MELD-Na score may be used to predict OS for patients undergoing pancreaticoduodenectomy due to periampullary cancer.

**Supplementary Information:**

The online version contains supplementary material available at 10.1007/s13304-024-01856-w.

## Introduction

Periampullary cancers (PAC) consist of four types of cancers as localized in the second continent of the duodenum, the ampulla of Vater, the head of the pancreas, and the distal common bile duct [1]. Most patients with PAC present with painless jaundice due to biliary obstruction. At the time of admission, patients are either unresectable or have distant metastases. The most preferred treatment method for operable patients is pancreaticoduodenectomy (PD). Dr. A. O. Whipple performed the first successful pancreaticoduodenectomy in 1934 [2]. The overall survival (OS) of patients undergoing PD for PAC is significantly longer than in nonoperative patients. The postoperative median OS in PACs was 9.8 months, regardless of location [3]. The 5-year survival was found to be 11% in pancreatic head adenocarcinoma and 39% in ampullary cancer [1]. Many studies investigated factors that affected OS in patients who underwent surgery for PAC. Many factors, such as age, CA 19–9, T-stage, N-stage, and adjuvant chemotherapy, were found to be effective in these studies [4, 5].

The model for end-stage liver disease (MELD) scoring was developed to calculate early mortality in patients with chronic liver disease [6]. It is calculated using creatine, international normalized ratio (INR), and bilirubin levels. The MELD-Na score was later developed by incorporating Na^+^ into the MELD score. This scoring is used to estimate 90-day mortality in chronic liver disease. MELD and MELD-Na scores have been reported to be associated with OS in patients with cirrhosis, hepatocellular carcinoma (HCC), and acid-developing pancreatic and gastric cancer [7–9]. However, the MELD-Na score has not been previously investigated for OS in patients with PAC undergoing PD.

This study aimed to investigate the prognostic effect of MELD-Na scores on OS, together with demographic and histopathologic data, in patients who underwent PD for PAC.

## Methods

### Ethical approval and patient selection

The study was started after receiving the approval of the ethics committee (Noninvasive Research Ethics Committee of Fırat University (approval no. 2023/05–15)) and the approval of the administration. Patients who underwent PD in the same clinic between January 2010 and January 2021 were included in the study. Patients aged 18 years or older who were diagnosed as having PAC and were accepted as resectable by the multidisciplinary oncology council (medical oncologist, radiologist, and surgical oncologist) were included in the study.

### Exclusion criteria

The exclusion criteria were as follows: benign and premalignant pathology, tumors other than adenocarcinomas, total or distal pancreatectomy, additional organ resection, metastatic disease, neoadjuvant therapy, known systemic immune disease, hematologic disease, chronic liver disease, chronic kidney disease, palliative surgical treatment, and missing data.

### Demographic and laboratory data

Data on age, sex, age-adjusted Charlson Comorbidity Index (ACCI), comorbidities (diabetes mellitus, pulmonary disease, heart disease, hepatitis), American Society of Anesthesiology scores (ASA), albumin, albumin/bilirubin ratio, neutrophil/lymphocyte ratio (NLR), CA 19–9 levels, MELD-Na score at admission, MELD-Na score at discharge, common bile duct and Wirsung canal diameters, and the presence of stents in the common bile duct were recorded. The OS of the patients was obtained from the hospital data system.

### Calculating MELD-Na score

MELD-Na = MELD-Na (mEq/L) – (0.025 × MELD ×  (140-Na)) + 140,

MELD(UNOS) = [[(0.957 × Ln creatinine (mg/dL)) + (0.378 × Ln bilirubin (mg/dL)) + (1.12 × Ln INR)] + 0.643] × 10.

MELD-unos = model for end-stage liver disease-UNOS (score).

(Na + is limited to a range of 125–140).

Entries < 1.0 are set to 1.0 for the purposes of the MELD score calculation.

Ln = natural logarithm.

UNOS = United Network for Organ Sharing.

### Surgical procedure

All patients underwent surgery at the same clinic. The retropancreatic area and portal vein were examined for resectability after a general exploration. For the surgical margin, resection was planned at the junction of all patients’ superior mesenteric and splenic veins. A frozen examination of the surgical margin was performed. Resection was mostly performed with pylorus resection (PRPD: pylorus-resected PD). Pyloric-sparing PD (PSPD) was performed in a limited number of patients with early-stage disease. Pancreaticojejunostomy (PJ) anastomoses were performed as duct-to-mucosa or dunking. Simultaneous porta resections were performed if needed. Surgery was terminated by routinely placing two drains at the surgical site.

### Peri–postoperative data

Peri–postoperative data of the patients, including T-stage, N-stage, lymphovascular invasion (LVI), surgical margin (R0: negative margin, R1: microscopically positive), tumor location, resection type, portal vein resection, perioperative blood loss, and postoperative pancreatic fistula (POPF) status, were recorded. T-stage was grouped as T1–2 and T3–4, and N-stage as N0 and N( +). Tumor locations were grouped as follows: the pancreatic head and distal common bile duct as one group, and the duodenum and ampulla as one group. Postoperative adjuvant treatment regimens were scanned via hospital data system and patients’ electronic pulse systems.

### Assessment of POPF and the study groups

POPF was evaluated and classified according to the International Study Group of Pancreatic Fistula classification. Drain amylase output in relation to the length of drain placement, endoscopic or percutaneous interventions, angiographic procedures, reoperation, length of hospital stay, organ failure, and death was assessed for classification. Grade A fistulas were assessed as POPF (–), and grade B and C fistulas were assessed as POPF ( +).

### Statistical analysis

All data were analyzed using the SPSS 22 statistic software package. The data were tested for normality of distribution using the Kolmogorov–Smirnov and Shapiro–Wilk tests. Parametric data are given as mean ± standard deviation (SD), and non-parametric data are given as median (minimum–maximum). Parametric data were evaluated using the independent samples *t* test, and non-parametric data were evaluated using the Mann–Whitney *U* test. The Chi-square or Fisher’s exact tests were used for categorical data. Pearson/Spearman correlation tests were used for correlation analyses. Cox regression analysis was used to determine the prognostic risk factors for OS. Covariates that showed significant associations with early mortality in univariate analysis (*p* < 0.1) were subjected to multivariate analysis. Multivariate analysis was performed using the Cox proportional hazard regression model (Forward LR method). The results are presented as hazard ratios (HR) with 95% confidence intervals (CIs). Overall survival was defined as the time between surgery and death and was censored at the last follow-up date if the patients were still alive. *P* values of < 0.05 were considered statistically significant.

## Results

### Demographic, laboratory and histopathologic data

Of the 124 patients, 80 patients who met the inclusion criteria were included in the study. The mean age of the patients was 61.74 ± 12.30 years. Thirty-one (38.8%) patients were female and 49 (61.2%) were male. The mean MELD-Na score at admission was 15.44 ± 5.56, MELD-Na score at discharge was 11.31 ± 3.59, and the mean albumin level was 3.85 ± 0.55 (g/dL). The median ACCI was 5 (range, 2–9), 0.57 (0.11–15) for the albumin/bilirubin ratio, 2.63 (0.81–46.19) for NLR, and 66.97 (0.01 to 41,404) (U/mL) for Ca 19–9. LVI ( +) was observed in 52 patients, ductal surgical margin ( +) in 4 patients, N ( +) in 43 patients, POPF ( +) in 5 patients, portal vein resection ( +) in 9 patients, and stents inserted in the choledochus in 62 patients (Table 1).

**Table 1:** Demographic and laboratory data of patients

Variables	*N* = 80 (%)
Age (years)	61.74 ± 12.30
Sex	
Male	49 (61.2%)
Female	31 (38.8%)
ASA score	
ASA II	8 (10%)
ASA III	68 (85%)
ASA IV	4 (5%)
ACCI	5 (2–9)
Heart diseases	
( +)	19 (23.7%)
(–)	61 (76.3%)
Pulmonary diseases	
( +)	17 (21.2%)
(–)	63 (78.8%)
Diabetes mellitus	
( +)	28 (35%)
(–)	52 (65%)
Hepatitis	
( +)	6 (7.5%)
(–)	74 (92.5%)
Tumor location	
Pancreatic head and distal choledochus	50 (62.5%)
Ampulla and duodenum	30 (37.5%)
T-stage	
T1–2	33 (41.2%)
T3–4	47 (58.8%)
N-stage	
N (0)	37 (46.3)
N ( +)	43 (53.7%)
Surgical margin	
R 0	76 (95%)
R 1	4 (5%)
LVI	
( +)	52 (65%)
(–)	28 (35%)
MELD-Na score at admission	15.44 ± 5.56
MELD-Na score at discharge	11.31 ± 3.59
Albumin (g/dL)	3.85 ± 0.55
Albumin/bilirubin ratio	0.57 (0.11–15)
NLR	2.63 (0.81–46.19)
CA 19–9 (U/mL)	66.97 (0.01–41,404)
POPF	
POPF ( +)	5 (6.3%)
POPF (–)	75 (93.7%)
Blood loss (mL)	475 (150–1200)
Choledochus diameter (mm)	14.64 ± 5.26
Wirsung diameter (mm)	4 (2–13)
Stent in choledochus	
Inserted	62 (77.5%)
No stent	18 (22.5%)
Resection type	
PRPD	55 (68.8%)
PPPD	25 (31.2%)
Portal vein resection	
Resected	9 (11.2%)
No resection	71 (88.8%)
Adjuvant treatment	
No treatment	16 (20%)
Regimen unknown	15 (18.7%)
5-FU-based regimens	17 (21.3)
Gemcitabine-based regimens	32 (40%)

### Relationship of MELD-Na score at admission

The MELD-Na score at admission was associated with biliary stent status, choledochus diameter, and survival time (*p* < 0.05). There was no correlation or significant difference found between the MELD-Na score and other pathological or laboratory data (Table 2).

**Table 2:** Relationship between MELD-Na score at admission and tumor characteristics and related parameters (* = correlation analysis performed (*r* = 0.349 for choledochus and *r* = –0.237 for survival))

Variables	MELD-Na score at admission	*p* value
T stage		
T 1–2	14.95 (2.97–25.94)	0.699
T 3–4	15.63 (3.72–33.85)	
N stage		
N (0)	15.39 (2.97–33.85)	0.689
N ( +)	15.33 (6.43–23.51)	
LVI		
(–)	14.64 (3.72–33.85)	0.280
( +)	15.83 (2.97–25.12)	
Location		
Pancreatic head and DC	15.73 (2.97–33.85)	0.366
Ampulla and duodenum	14.86 (6.43–25.94)	
Biliary stenting		
Inserted	15.88 (3.72–33.85)	**0.015**
None	12.60 (2.97–22.32)	
Choledochus diameter		0.002*
Wirsung diameter		0.185*
Albumin		0.071*
Albumin/bilirubin ratio		0.871*
CA 19–9		0.378*
Survival (months)		0.034*

*p* values that are significant are accentuated in bold and italic within the table

At the start of the study, 24 patients were alive, if all patients were classified as dead or alive. There was a significant difference between the groups in terms of albumin level (*p* = 0.048)(Supplementary table). There was no significant difference between the groups in terms of other variables (*p* > 0.05).

### Univariate and multivariate analyses

Univariate and multivariate analyses of the predictive factors associated with OS are presented in Table 3. Univariate analysis showed that the MELD-Na scores at admission (HR: 1.061, 95% CI [1.011–1.113]; *p* = 0.016), ACCI (HR: 1.173, 95% CI [1.012–1.360]; *p* = 0.034), adjuvant treatment (HR: 3.90, 95% CI [2.104–7.232]; *p* < 0.001), LVI (HR: 1.733, 95% CI [0.972–3.089]; *p* = 0.062), tumor location (HR: 2.022, 95% CI [1.138–3.593]; *p* = 0.016), T-stage (HR: 1.715, 95% CI [0.986–2.984]; *p* = 0.056), and portal vein resection (HR: 1.997, 95% CI [0.939–4.246]; *p* = 0.072) were significantly associated with OS(*p* < 0.1). Variables that had significant levels in univariate analysis (*p* < 0.1) were entered into the multivariate Cox proportional hazards model, and the results indicated that MELD-Na score at admission (HR: 1.051, 95% CI [1.004–1.101]; *p* = 0.033), adjuvant treatment (HR: 4.717, 95% CI [2.371–9.383]; *p* < 0.001), LVI (HR: 2.473, 95% CI [1.355–4.515]; *p* = 0.003), and tumor location (HR: 2.380, 95% CI [1.274–4.445]; *p* = 0.007) were independent risk factors for OS (*p* < 0.05).

**Table 3:** Univariate and multivariate analyses of prognostic factors for overall survival

Variables	Univariate analyses		Multivariate analyses	
	HR 95% CI	*p* value	HR 95% CI	*p* value
Age	1.012 (0.988–1.036)	0.338		
Sex	1.033 (0.604–1.766)	0.906		
ASA				
II		0.654		
III	0.761(0.342–1.693)	0.503		
IV	0.540 (0.138–2.108)	0.375		
ACCI	1.173 (1.012–1.360)	**0.034**		
Adjuvant treatment	3.90 (2.104–7.232)	** < 0.001**	4.717 (2.371–9.383)	** < 0.001**
MELD-Na score at admission	1.061 (1.011–1.113)	**0.016**	1.051 (1.004–1.101)	**0.033**
MELD-Na score at discharge	1.008 (0.936–1.084)	0.840		
Albumin	1.066 (0.622–1.827)	0.816		
Albumin/bilirubin ratio	0.926 (0.842–1.019)	0.115		
NLR	1.022 (0.984–1.061)	0.267		
CA 19–9	1 (1–1)	0.342		
Biliary stenting	1.106 (0.569–2.152)	0.766		
Resection type	1.148 (0.661–1.995)	0.623		
Porta resection	1.997 (0.939–4.246)	**0.072**		
Blood loss	1 (0.999–1.001)	0.849		
Choledochus diameter	0.966 (0.922–1.012)	0.142		
Wirsung diameter	1.054 (0.951–1.168)	0.317		
T-stage	1.715 (0.986–2.984)	**0.056**		
N-stage	1.421 (0.830–2.432)	0.200		
LVI	1.733 (0.972–3.089)	**0.062**	2.473 (1.355–4.515)	**0.003**
Surgical margin	0.792 (0.283–2.223)	0.658		
Location	2.022 (1.138–3.593)	**0.016**	2.380 (1.274–4.445)	**0.007**

*p* values that are significant are accentuated in bold and italic within the table

**Fig. 1:**
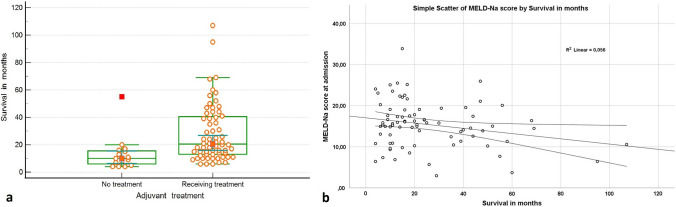
The graphics of the association of OS with the MELD-Na score and adjuvant treatment (**a:** boxplot diagram of adjuvant treatment groups, **b:** MELD-Na score (*p* = 0.034 *r* = –0.237))

The MELD-Na score at admission was associated with OS (*p* = 0.034 *r* = –0.237) and the OS was longer in the adjuvant treatment received group (Fig. 1).

## Discussion

In this study, the MELD-Na scores at admission, adjuvant treatment, LVI, and tumor location were found to be independent risk factors for OS in patients undergoing PD for PAC. This is the first study to investigate the MELD-Na score as a risk factor for OS in PAC patients undergoing PD.

The MELD-Na score is used to predict 90-day mortality in patients with chronic liver disease. This scoring has been investigated for OS in patients with HCC, chronic hepatitis B, and ascites developing in pancreatic and gastric cancer [7, 8, 10]. In these studies, the MELD-Na score was associated with OS. In our study, the MELD-Na score was found to be a prognostic risk factor and associated with OS in patients undergoing PD due to PAC. The association of MELD-Na scores with OS may be due to the effect of biliary obstruction on the liver, kidney, immune system, and cardiovascular system (CVS) [11].

Biliary obstruction causes dysfunction in the immune system [11–13]. Endotoxins and hyperbilirubinemia may act on T cells and cause a decrease in the number of CD4^+^ T cells. The decrease in the CD4^+^ T cell ratio in patients with biliary obstruction compared with patients with non-obstructive disease may cause a decrease in OS because it may cause cancer progression [12].

Biliary obstruction causes an increase in serum bile acids and bilirubin levels. Increased bile acids may bind to some receptors and cause undesirable effects in the CVS. These receptors can be nuclear or membrane receptors, as well as Ca^+^ activating K^+^ channels. These receptors have been observed in cardiac myocytes, fibroblasts, and vascular smooth muscle cells. Increased bile acids may have effects such as bradycardia or decreased vascular resistance [14]. These negativities may lead to a decrease in OS over CVS.

Another effect of biliary obstruction is hyponatremia and increased atrial natriuretic peptide (ANP) levels. In a study, Na^+^ levels were found to be associated with OS in patients with biliary tree cancer [15]. Low Na^+^ levels are associated with short OS. ANP levels were found to be increased in experimentally created biliary ligation and biliovenous shunt groups [16]. This increase indirectly causes hyponatremia. In addition, hyponatremia was found to be associated with ANP in patients with chronic liver disease [17]. The MELD-Na score increases with hyponatremia. In our study, high MELD-Na scores were found to be associated with short OS. Our study shows similar results to the literature.

Comorbidities may increase with age. It is possible to experience a decrease in OS as comorbidity increases. For this reason, Mary Charlson created the Charlson Comorbidity Index (CCI) index in 1987 [18]. Later, age was added to this index, and the ACCI began to be used [19]. ACCI has been studied previously in pancreatic and ampulla cancer and has been associated with OS [20, 21]. In our study, ACCI was found to be a risk factor associated with OS in univariate analysis. However, it was not statistically significant in multivariate analysis. This status may be due to the study’s design. In the present study, multivariate analysis included the patients who both received adjuvant treatment and did not.

T-stage is part of the TNM staging used in most cancers. The effect of TNM staging on prognosis in PAC has been investigated in many studies [5, 22–26]. In these studies, there is a decrease in OS as T-stage and/or N-stage increase. In the present study, T-stage and N-stage were found to be insignificant in multivariate analysis.

LVI, which is one of the prognostic factors that affect OS, has been shown in many studies to be a poor prognostic factor [27, 28]. However, there are also studies reporting that LVI is unrelated to OS [22, 29]. Different results may be due to different prognostic factors in the analysis. In our study, LVI was found to be significant in multivariate analysis. Patients with a positive LVI had a shorter OS.

The target should be R0 resection in patients who undergo surgery for malignancies. This is important for local recurrence and OS. In studies for PAC, R1 resection has been indicated as an important prognostic factor for OS [5, 30]. However, Vilhordo et al. reported that R1 resection was not associated with OS [27]. In our study, surgical margins were found to be nonsignificant for OS.

In PACs, the shortest OS is observed in the pancreas and the longest in the duodenum (pancreas, DC, ampulla, and duodenum, respectively) [1]. This may be due to the nature of the tumor, which arises from a pancreatobiliary or intestinal subtype. As reported in the literature, intestinal-type ampullary carcinomas have a longer OS than pancreatobiliary types [31, 32]. In the present study, tumor location was found to be an independent risk factor for OS. Tumors in the pancreatic head and DC have a shorter OS than duodenal and ampullary cancers.

Adjuvant treatment is recommended after PD due to the PAC. In studies, OS is longer in the adjuvant treatment group [32, 33]. On the contrary, there are also studies reporting that it is not effective [34, 35]. The reason for this controversy is probably the subtypes of PACs and other prognostic factors [32, 36, 37]. Studies recommend individual treatments, discussed by a multidisciplinary team [38]. In the present study, adjuvant treatment was found to be an independent risk factor for OS. Patients receiving adjuvant therapy had a longer OS.

Our limitations are the limited number of patients, the retrospective nature of the study, the inability to access a limited number of chemotherapy regimens, the absence of tumor subtypes, and the absence of data on noncancer-related deaths.

In conclusion, MELD-Na score at admission, adjuvant treatment, LVI, and tumor location were found to be independent risk factors associated with OS in patients undergoing PD for PAC. We think that the association of the MELD-Na score with OS might be related to effects secondary to biliary obstruction. Based on our findings, the MELD-Na score may be a useful marker to predict OS in patients with PAC.

## Supplementary Information

Below is the link to the electronic supplementary material.

Supplementary file1 (DOCX 17 KB)
