# Supplementary material for: Effect of MELD-Na score on overall survival of periampullary cancer
Source: Updates Surg. 2024 May 7;76(5):1819–25. doi: 10.1007/s13304-024-01856-w (PMC11455662; doi:10.1007/s13304-024-01856-w)
Supplement: Supplementary file 1 — Supplementary file1 (DOCX 17 KB) [file 13304_2024_1856_MOESM1_ESM.docx]

**Supplementary table:** Differences between the alive and exitus groups (The status of the patients includes their status at the beginning of the study. There is no time point).

| **Variables** | | **Alive group**  **n: 24** | **Exitus group**  **n: 56** | ***p-value*** |
| --- | --- | --- | --- | --- |
| Survival (months) | | 20 (6-95) | 16.5 (4-107) | 0.122 |
| Age (years) |  | 64.14 ± 10.71 | 60.67 ± 13.07 | 0.633 |
| Sex | Male | 17 | 32 | 0.249 |
|  | Female | 7 | 24 |  |
| ASA score | ASA II | 1 | 7 | 0.672 |
|  | ASA III | 22 | 46 |  |
|  | ASA IV | 1 | 3 |  |
| ACCI | | 5 (2-7) | 5 (2-9) | 0.762 |
| Tumor location | Pancreatic head and DC | 15 | 35 | 1.00 |
|  | Ampulla and duodenum | 9 | 21 |  |
| T-stage | T1-2 | 12 | 21 | 0.298 |
|  | T3-4 | 12 | 35 |  |
| N-stage | N 0 | 13 | 24 | 0.353 |
|  | N (+) | 11 | 32 |  |
| Surgical margin | R 0 | 24 | 52 | 0.311 |
|  | R 1 | 0 | 4 |  |
| LVI | (+) | 15 | 37 | 0.795 |
|  | (-) | 9 | 19 |  |
| Preoperative  MELD-Na score | | 13.86 ± 5.78 | 16.43 ± 5.60 | 0.078 |
| Postoperative  MELD-Na score | | 11.06 ± 3.89 | 11.31 ± 3.88 | 0.944 |
| Albumin (g/dL) | | 3.01 ± 0.45 | 3.81 ± 0.58 | **0.048** |
| Albumin/Bilirubin ratio | | 0.59 (0.27-14.67) | 0.56 (0.11-15) | 0.156 |
| NLR | | 3.16 (1.45-12.16) | 2.45 (0.81-46.19) | 0.996 |
| CA 19-9 (U/mL) | | 47.9 (0.01-10413) | 75 (1.17-41404) | 0.895 |
| POPF | POPF (+) | 1 | 4 | 0.1 |
|  | POPF (-) | 23 | 52 |  |
| Blood loss (mL) | | 400 (150-900) | 500 (150-1200) | 0.214 |
| Choledochus diameter (mm) | | 15 (5-21) | 14 (4-27) | 0.200 |
| Wirsung diameter (mm) | | 3 (2-9) | 4 (2-13) | 0.581 |
| Stent in choledochus | Inserted | 17 | 45 | 0.389 |
|  | No stent | 7 | 11 |  |
| Resection type | PRPD | 19 | 36 | 0.292 |
|  | PPPD | 5 | 20 |  |
| Portal vein resection | Resected | 1 | 8 | 0.266 |
|  | No resection | 23 | 48 |  |
